# Evaluation and Mitigation of Racial Bias in Clinical Machine Learning Models: Scoping Review

**DOI:** 10.2196/36388

**Published:** 2022-05-31

**Authors:** Jonathan Huang, Galal Galal, Mozziyar Etemadi, Mahesh Vaidyanathan

**Affiliations:** 1 Department of Anesthesiology Northwestern University Feinberg School of Medicine Chicago, IL United States; 2 Department of Biomedical Engineering Northwestern University Evanston, IL United States; 3 Digital Health & Data Science Curricular Thread Northwestern University Feinberg School of Medicine Chicago, IL United States

**Keywords:** artificial intelligence, machine learning, race, bias, racial bias, scoping review, algorithm, algorithmic fairness, clinical machine learning, medical machine learning, fairness, assessment, model, diagnosis, outcome prediction, score prediction, prediction, mitigation

## Abstract

**Background:**

Racial bias is a key concern regarding the development, validation, and implementation of machine learning (ML) models in clinical settings. Despite the potential of bias to propagate health disparities, racial bias in clinical ML has yet to be thoroughly examined and best practices for bias mitigation remain unclear.

**Objective:**

Our objective was to perform a scoping review to characterize the methods by which the racial bias of ML has been assessed and describe strategies that may be used to enhance algorithmic fairness in clinical ML.

**Methods:**

A scoping review was conducted in accordance with the Preferred Reporting Items for Systematic Reviews and Meta-analyses (PRISMA) Extension for Scoping Reviews. A literature search using PubMed, Scopus, and Embase databases, as well as Google Scholar, identified 635 records, of which 12 studies were included.

**Results:**

Applications of ML were varied and involved diagnosis, outcome prediction, and clinical score prediction performed on data sets including images, diagnostic studies, clinical text, and clinical variables. Of the 12 studies, 1 (8%) described a model in routine clinical use, 2 (17%) examined prospectively validated clinical models, and the remaining 9 (75%) described internally validated models. In addition, 8 (67%) studies concluded that racial bias was present, 2 (17%) concluded that it was not, and 2 (17%) assessed the implementation of bias mitigation strategies without comparison to a baseline model. Fairness metrics used to assess algorithmic racial bias were inconsistent. The most commonly observed metrics were equal opportunity difference (5/12, 42%), accuracy (4/12, 25%), and disparate impact (2/12, 17%). All 8 (67%) studies that implemented methods for mitigation of racial bias successfully increased fairness, as measured by the authors’ chosen metrics. Preprocessing methods of bias mitigation were most commonly used across all studies that implemented them.

**Conclusions:**

The broad scope of medical ML applications and potential patient harms demand an increased emphasis on evaluation and mitigation of racial bias in clinical ML. However, the adoption of algorithmic fairness principles in medicine remains inconsistent and is limited by poor data availability and ML model reporting. We recommend that researchers and journal editors emphasize standardized reporting and data availability in medical ML studies to improve transparency and facilitate evaluation for racial bias.

## Introduction

### Background

In recent years, artificial intelligence (AI) has drawn significant attention in medicine as machine learning (ML) techniques show an increasing promise of clinical impact. Driven by unprecedented data accessibility and computational capacity, ML has been reported to reach parity with human clinicians in a variety of tasks [1-3]. ML is poised to benefit patients and physicians by optimizing clinical workflows, enhancing diagnosis, and supporting personalized health care interventions [4-6]. Decision support tools based on ML have already been implemented across health systems [7,8], and the continued proliferation of clinical ML will impact patients in all fields of medicine.

However, despite its appeal, significant barriers remain to the full realization of clinically integrated ML. Key concerns include limited model transparency due to the “black box” of ML, inadequate reporting standards, and the need for prospective validation in clinical settings [1,9-12]. Racial bias in clinical ML is a crucial challenge arising from these limitations and must be addressed to ensure fairness in clinical implementation of ML. As ML is premised on prediction of novel outcomes based on previously seen examples, unintended discrimination is a natural consequence of algorithm development involving training data that reflect real-world inequities [13].

Equity in health care remains a continual pursuit [14,15]. Bias and disparities along dimensions of race, age, and gender have been shown to impact health care access and delivery, evident in varied settings, such as race correction in clinical algorithms or clinical trial enrollment and adverse event monitoring [16,17]. Considering the growing body of literature demonstrating profound adverse impacts of health care inequities on patient outcomes, mitigation of the numerous and insidious sources of potential bias in medicine requires remains a critical challenge to prevent harm to patients [14,17]. Thus, the potential for algorithms to perpetuate health disparities must be carefully weighed when incorporating ML models into clinical practice [18-20].

Algorithmic fairness is an area of ML research guiding model development with the aim of preventing discrimination involving protected groups, which are defined by attributes such as race, gender, religion, physiologic variability, preexisting conditions, physical ability, and sexual orientation [13,19]. However, application of algorithmic fairness principles in the medical ML literature remains nascent [20]. Greater awareness of the potential harms of bias in clinical ML as well as methods to evaluate and mitigate them is needed to support clinicians and researchers across the health care and data science disciplines, who must evaluate and implement clinical ML models with a critical eye toward algorithmic fairness. The objective of this study is to characterize the impact and mitigation of racial bias in clinical ML to date and describe best practices for research efforts extending algorithmic fairness to medicine.

### Bias and Fairness in Machine Learning

**Figure 1:**
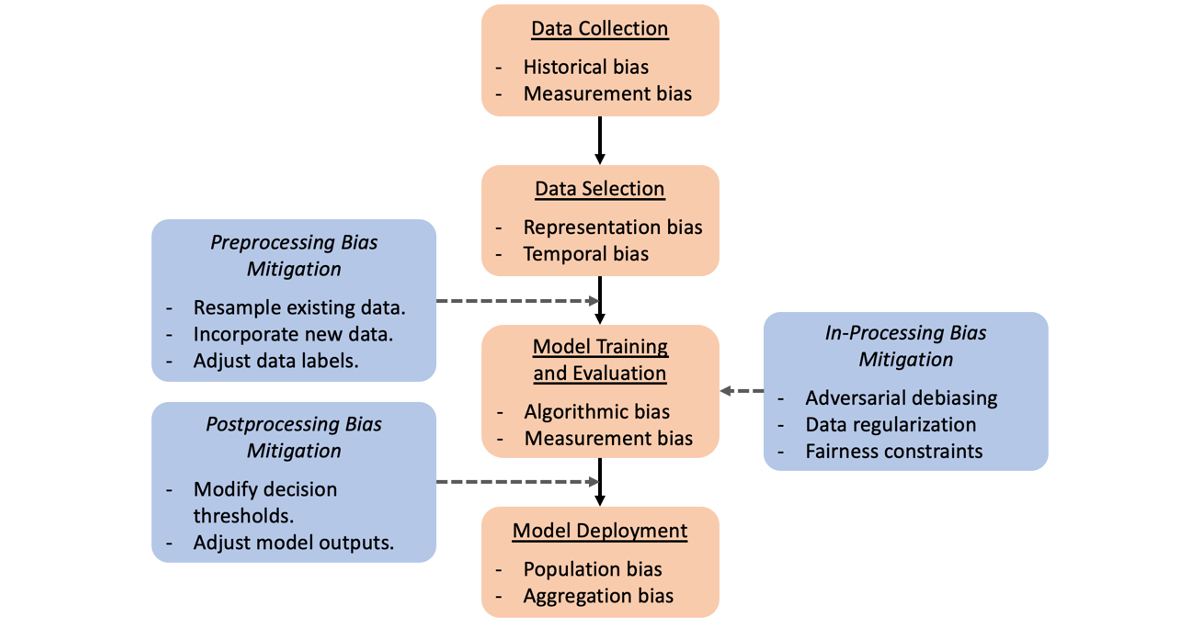
The clinical machine learning development workflow (orange boxes) offers several opportunities (blue boxes) to evaluate and mitigate potential biases introduced by the data set or model. Preprocessing methods seek to adjust the existing data set to preempt biases resulting from inadequate data representation or labeling. In-processing methods impose fairness constraints as additional metrics optimized by the model during training or present data in a structured manner to avoid biases in the sampling process. Postprocessing methods account for model biases by adjusting model outputs or changing the way they are used.

In the setting of algorithmic fairness, bias is present when an algorithm systematically favors one outcome over another. Bias may be introduced into an ML algorithm throughout all steps of the development process, which involves data collection, data selection, model training, and model deployment [13]. Examples of these sources of bias are shown in Figure 1, and their definitions are given in Multimedia Appendix 1. Notably, historical bias may be present even if all steps of model development are optimally performed. This is of particular concern in the evaluation of racial bias in clinical ML, given the presence of existing and historical health care disparities [14].

Depending on the context, bias in clinical ML may not be harmful and can even be used to overcome inequality [13]. In situations in which targeting a well-defined subpopulation above all others is desirable, an ML algorithm biased toward a particular group may be used to proactively mitigate existing disparities. However, bias may arise when ML models designed to serve the needs of a specific clinical population—such as a particular community or high-risk demographic—are inappropriately applied to other populations or when more general models are applied to specific populations. Additionally, ML algorithms tend to overfit to the data on which they are trained, which entails the learning of spurious relationships present in the training data set and may result in a lack of generalizability to other settings. As a result, a model that appears unbiased in one setting may display bias in another. Thus, bias in clinical ML must be considered in the light of the context and particular population of interest.

Bias in an ML model may lead to unfairness if not appropriately evaluated and accounted for. Fairness in ML is achieved when algorithmic decision-making does not favor an individual or group based on protected attributes. Research efforts have emphasized group fairness over individual fairness, given the need for algorithms that consider existing differences between populations—whether intrinsic or extrinsic—while preventing discrimination between groups [13,21]. Crucially, improving model fairness does not necessarily require compromising accuracy overall [22]. For instance, an unfair disease-screening tool might have poor sensitivity for disease detection in one low-risk population subgroup compared to another with higher risk; improving the fairness of this tool would entail adjusting the model to have more similar sensitivities between subgroups. In this study, we examine the racial bias of clinical ML in terms of model fairness with respect to race.

### Assessing and Achieving Fairness in Machine Learning

Group fairness is quantified by evaluating the similarity of a given statistical metric between predictions made for different groups. Group fairness indicators encountered in this review are defined in Table 1. Critical examinations of different methods for evaluating fairness in ML, both in general application [13,23,24] and in the context of health care [21], have been previously described, though applications in clinical ML remain limited. It is important to note that fairness metrics may be at odds with one another, depending on the context and application [25]; thus, evaluation of an appropriate metric, given the clinical situation of interest, is paramount [26].

Approaches to bias mitigation fall into 3 major categories (Figure 1): *preprocessing*, in which inequities in data are removed prior to model training; *in-processing*, in which the model training process is conducted to actively prevent discrimination; and *postprocessing*, in which outputs of a trained model are adjusted to achieve fairness [13]. Preprocessing can be performed by resampling existing data, incorporating new data, or adjusting data labels. In-processing methods use adversarial techniques, impose constraints and regularization, or ensure fairness of underlying representations during training. Finally, postprocessing entails group-specific modification of decision thresholds or outcomes to ensure fairness in the application of model predictions. Different approaches may be optimal depending on the setting and stage of model development.

**Table 1:** Group fairness metrics encountered in this review.

Term	Description
AUROC^a^	Assesses overall classifier performance by measuring the TPR^b^ and FPR^c^ of a classifier at different thresholds.
Average odds	Compares the average of the TPR and FPR for the classification outcome between protected and unprotected groups.
Balanced accuracy	A measure of accuracy corrected for data imbalance, calculated as the average of sensitivity and specificity for a group.
Calibration	Assesses how well the risk score or probability predictions reflect actual outcomes.
Disparate impact	Measures deviation from statistical parity, calculated as the ratio of the rate of the positive outcome between protected and unprotected groups. Ideally, the disparate impact is 1.
Equal opportunity	For classification tasks in which one outcome is preferred over the other, equal opportunity is satisfied when the preferred outcome is predicted with equal accuracy between protected and unprotected groups. Ideally, the TPR or FNR^d^ disparity between groups is 0.
Equalized odds	The TPR and FPR are equal between protected and unprotected groups.
Error rate	Compares the error rate of predictions, calculated as the number of incorrect predictions divided by the total number of predictions, between protected and unprotected groups. Ideally, the error rate disparity between groups is 0.
Statistical parity	Statistical parity (also known as demographic parity) is satisfied when the rate of positive outcomes is equal between protected and unprotected groups.

^a^AUROC: area under the receiver operating characteristic curve.

^b^TPR: true-positive rate.

^c^FPR: false-positive rate.

^d^FNR: false-negative rate.

## Methods

### Study Design

We performed a scoping review of racial bias and algorithmic fairness in clinical ML models in accordance with the Preferred Reporting Items for Systematic Reviews and Meta-analyses (PRISMA) 2020 guidelines [27] and PRISMA Extension for Scoping Reviews [28]. The review protocol was not registered and is available upon request to the authors. The PubMed MEDLINE (National Library of Medicine), Scopus (Elsevier), and Embase (Elsevier) databases were queried by combining terminology pertaining to ML, race, and bias as keywords. Additional records were identified using Google Scholar search. The exact search strategy is detailed in Multimedia Appendix 1.

### Study Selection

After duplicate record removal, studies were initially screened by title and abstract and then screened for final inclusion by full text review. All screening was performed independently by 2 reviewers. Studies were selected based on the following inclusion criteria: peer-reviewed original research, English language, full text available, development or evaluation of a clinically relevant ML model, and evaluation of bias of the model regarding racial or ethnic groups. Studies other than full-length papers were excluded. ML was defined as a computer algorithm that improves automatically via training on data [4]. Per PRISMA guidelines, any disagreements regarding study inclusion based on these criteria were reconciled by discussion.

### Data Abstraction

Relevant data were abstracted from included papers by 1 reviewer. Data of interest included the clinical objective of ML models, identification of racial bias, impact of racial bias, metrics for bias assessment, mitigation of racial bias, methods for bias mitigation, data set size, data source, ML model architecture, and availability of computer code used for data preparation and ML model development. The methodological quality of included studies was not assessed, given the scoping nature of this review [28].

## Results

### Study Characteristics

**Figure 2:**
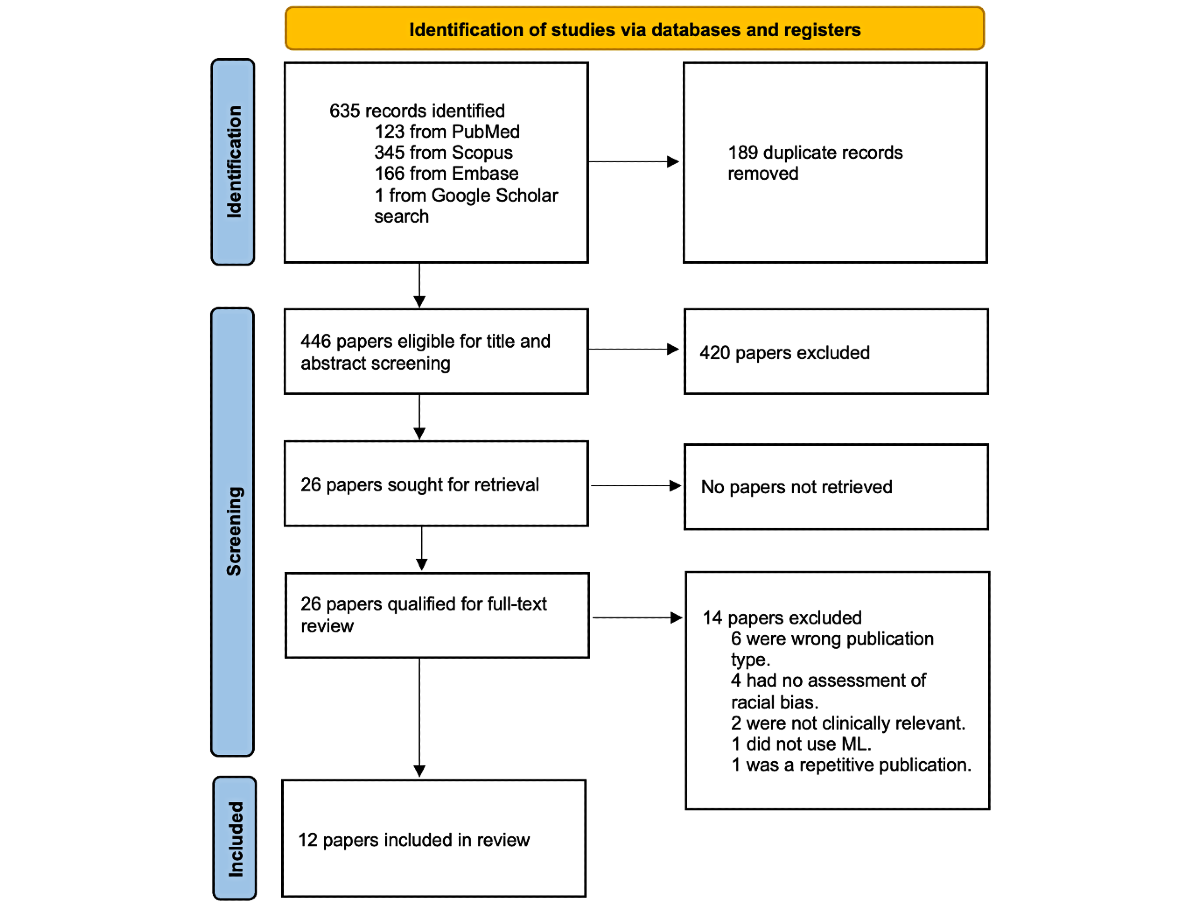
PRISMA flowchart of study inclusion. ML: machine learning; PRISMA: Preferred Reporting Items for Systematic Reviews and Meta-analyses.

The literature search was performed on September 8, 2021, and identified 635 records (Figure 2). Of these, 26 (4.1%) full-text papers were reviewed and 12 (46.2%) were included in the final analysis [29-40].

Characteristics of the included studies are summarized in Table 2. Data sets and models used are summarized in Multimedia Appendix 1. Of the 12 studies, 3 (25%) were published in 2019, 5 (42%) in 2020, and 4 (33%) in 2021. In addition, 9 (75%) studies originated from the United States, 1 (8%) from Canada, 1 (8%) from Sweden, and 1 (8%) from both the United Kingdom and Nigeria. Applications of ML were varied and involved diagnosis, outcome prediction, and clinical score prediction performed on data sets including images, diagnostic studies, clinical text, and clinical variables. Furthermore, 1 (8%) study described a model in routine clinical use [36], 2 (17%) examined prospectively validated clinical models [35,39], and the remaining 9 (75%) described internally validated models.

Of the 12 studies, 5 (42%) published code used for analysis, 3 (25%) made model development code available [34,36,39], 2 (17%) published bias analysis code [33,36], 1 (8%) published code relevant to debiasing [30], and 1 (8%) published data selection code [33]. In addition, 1 (8%) study used publicly available code for analysis [31], and code was specified as available upon request in 1 (8%) study [35]. Bias of an ML model was evaluated using an external database in 8 (67%) studies [30-34,37,38], single-institutional data in 3 (25%) studies [35,36,40], and data from 2 institutions in 2 (17%) studies [29,39]. No institutional data sets were published. Convolutional neural networks (CNNs) were the predominant ML modeling technique used (5/12, 42%), followed by logistic regression (3/12, 25%), least absolute shrinkage and selection operator (LASSO; 2/12, 17%), and extreme gradient boosting (XGBoost; 2/12, 17%). In addition, 3 (25%) studies evaluated models adapted from existing neural network architectures: ResNet50 in 2 (17%) studies [29,32] and DenseNet in the other [38].

Of the 12 studies, 9 (75%) evaluated a model developed internally by the same researchers [29-33,35,37,39,40], 2 (17%) evaluated a model developed externally by separate researchers [36,38], and 1 (8%) evaluated both internally and externally developed models [34]. In addition, 8 (67%) studies concluded that racial bias was present [29,32-34,36-39], 2 (17%) concluded that bias was not present [35,40], and 2 (17%) assessed the implementation of bias mitigation strategies without comparison to a baseline model [30,31]. A variety of methods were used to assess the presence of algorithmic racial bias: 3 (25%) studies used multiple metrics to assess fairness [31,34,37], while the remaining 9 (75%) used a single metric. The most commonly used fairness metrics were equal opportunity difference [41], defined either as the difference in the true-positive rate (TPR) or the false-negative rate (FNR) between subgroups (5/12, 42%) [30,31,38,39]; accuracy (4/12, 25%) [29,31,32,34]; and disparate impact (2/12, 17%) [31,37].

The approaches and efficacy of bias mitigation methods used in the studies evaluated are summarized in Table 3. All 8 (67%) studies that implemented methods for mitigation of racial bias successfully increased fairness, as measured by the authors’ chosen metrics [29-32,34,36,37,39]. Preprocessing bias mitigation was the most commonly used strategy (7/13, 54%). In addition, 1 (8%) study removed race information from the training data, though superior improvements in disparate impact and equal opportunity difference were achieved by reweighing [37]. Furthermore, 2 (17%) studies performed in-processing bias mitigation using the prejudice remover regularizer [42] or adversarial debiasing during model training [31,37]. However, in both studies, in-processing was ineffective in reducing bias and was outperformed by other bias mitigation methods. Finally, 1 (8%) study evaluated multiple types of ML models for bias during the development process, concluding that a LASSO model was preferable to conditional random forest, gradient boosting, and ensemble models for racially unbiased dementia ascertainment [34].

**Table 2:** Study characteristics.

Author (year)	Clinical objective	How was fairness evaluated?	Was racial bias identified?	How was the AI^a^ model biased?	Was racial bias mitigated?	Protected class
Abubakar et al (2020) [29]	Identification of images of burns vs healthy skin	Accuracy	Yes	Poor accuracy of models trained on a Caucasian data set and validated on an African data set and vice versa	Yes	Dark-skinned patients, light-skinned patients
Allen et al (2020) [30]	Intensive care unit (ICU) mortality prediction	Equal opportunity difference (FNR^b^ disparity)	N/A^c^	N/A	Yes	Non-White patients
Briggs and Hollmén (2020) [31]	Prediction of future health care expenditures of individual patients	Balanced accuracy, statistical parity, disparate impact, average odds, equal opportunity	N/A	N/A	Yes	Black patients
Burlina et al (2021) [32]	Diagnosis of diabetic retinopathy from fundus photography	Accuracy	Yes	Lower diagnostic accuracy in darker-skinned individuals compared to lighter-skinned individuals	Yes	Dark-skinned patients
Chen et al (2019) [33]	ICU mortality prediction, psychiatric readmission prediction	Error rate (0-1 loss)	Yes	Differences in error rates in ICU mortality between racial groups	No	Non-White patients
Gianattasio et al (2020) [34]	Dementia status classification	Sensitivity, specificity, accuracy	Yes	Existing algorithms varying in sensitivity and specificity between race/ethnicity groups	Yes	Hispanic, non-Hispanic Black patients
Noseworthy et al (2020) [35]	Prediction of left ventricular ejection fraction ≤35% from the electrocardiogram (ECG)	AUROC^d^	No	N/A	No	Non-White patients
Obermeyer et al (2019) [36]	Prediction of future health care expenditures of individual patients	Calibration	Yes	Black patients with a higher burden than White patients at the same algorithmic risk score	Yes	Black patients
Park et al (2021) [37]	Prediction of postpartum depression and postpartum mental health service utilization	Disparate impact, equal opportunity difference (TPR^e^ disparity)	Yes	Black women with a worse health status than White women at the same predicted risk level	Yes	Black patients
Seyyed-Kalantari et al (2021) [38]	Diagnostic label prediction from chest X-rays	Equal opportunity difference (TPR disparity)	Yes	Greater TPR disparity in Hispanic patients	No	Non-White patients
Thompson et al (2021) [39]	Identification of opioid misuse from clinical notes	Equal opportunity difference (FNR disparity)	Yes	Greater FNR in the Black subgroup than in the White subgroup	Yes	Black patients
Wissel et al (2019) [40]	Assignment of surgical candidacy score for patients with epilepsy using clinical notes	Regression analysis of the impact of the race variable on the candidacy score	No	N/A	No	Non-White patients

^a^AI: artificial intelligence.

^b^FNR: false-negative rate.

^c^N/A: not applicable.

^d^AUROC: area under the receiver operating characteristic curve.

^e^TPR: true-positive rate.

**Table 3:** Bias mitigation methods among reviewed studies.

Description of strategies used		Effectiveness
**Preprocessing**		
	Reweighing training data	An equal opportunity difference (FNR^a^ difference) of 0.016 (*P*=.20) was achieved for intensive care unit (ICU) mortality prediction [33]. The mean fairness measure (average of statistical parity difference, disparate impact measure, average odds difference, and equal opportunity difference) improved to 0.06 from 0.12 for prediction of health care costs [34]. Disparate impact improved from 0.31 to 0.79, and the equal opportunity (TPR^b^) difference improved from –0.19 to 0.02 for prediction of postpartum depression development; prediction of mental health service use in pregnant individuals improved from 0.45 to 0.85 and –0.11 to –0.02, respectively [40].
	Combining data sets to increase heterogeneity	The accuracy of skin burn identification increased to 99.5% using a combined data set compared to 83.4% and 87.5% when trained on an African and evaluated on a Caucasian data set and vice versa [32].
	Generating synthetic minority class data	Disparity in diabetic retinopathy diagnostic accuracy improved from 12.5% to 7.5% and 0.5% when augmenting with retina appearance-optimized images and diabetic retinopathy status-optimized images created with a generative adversarial network, respectively [35].
	Adjusting label selection	Improved congruence in health outcomes between groups after developing models to predict other labels for health status besides financial expenditures [39].
	Removing race information from training data	Disparate impact improved from 0.31 to 0.61 and equal opportunity (TPR) difference improved from –0.19 to –0.05 for prediction of postpartum depression development; respective improvements from 0.45 to 0.63 and –0.11 to –0.04 for prediction of mental health service use in pregnant individuals [40].
**In-processing**		
	Use of a regularizer during training	Disparate impact improved, but accuracy and the equal opportunity (TPR) difference decreased when implementing the prejudice remover regularizer in prediction of postpartum depression in pregnant individuals [40].
	Adversarial debiasing	The mean fairness measure (average of statistical parity difference, disparate impact measure, average odds difference, and equal opportunity difference) worsened to 0.07 from 0.05 for prediction of health care costs [34].
**Postprocessing**		
	Calibration	The equal opportunity (FNR) difference improved from 0.15 to 0.03 for identification of opioid misuse [42].
	Reject option-based classification	The mean fairness measure (average of statistical parity difference, disparate impact measure, average odds difference, and equal opportunity difference) improved to 0.09 from 0.15 for prediction of health care costs [34].
	Varying cut-point selection	The equal opportunity (FNR) difference improved from 0.15 to 0.04 for identification of opioid misuse [42]. The congruence in sensitivity and specificity between groups improved without reduction in accuracy for classification of dementia status [37].

^a^FNR: false-negative rate.

^b^TPR: true-positive rate.

## Discussion

### Principal Findings

Given the pressing issue of equity in health care and the rapid development of medical ML applications, racial bias must be thoroughly evaluated in clinical ML models in order to protect patient safety and prevent the algorithmic encoding of inequality. Algorithmic fairness is a relatively novel field within the discipline of ML, and its application to medical ML remains nascent. In our evaluation of the literature describing mitigation of racial bias in clinical ML, we identified a variety of bias mitigation methods, which when applied successfully increase fairness and demonstrate the feasibility and importance of racial bias evaluation in the medical ML development process. Based on our findings, there is a need for heightened awareness of algorithmic fairness concepts, increased data availability, and improved reporting transparency in medical ML development to ensure fairness in clinical ML.

### Impact of Racial Bias in Clinical Machine Learning

The broad scope of medical ML applications and potential patient harms following deployment across health care systems demand an increased emphasis on evaluation and mitigation of racial bias in clinical ML. Screening and outcome prediction tasks are commonly examined among reviewed studies. Racial bias in such tasks is particularly concerning as decisions made from flawed models trained on data, which reflect historical inequities in disease diagnosis and care delivery, may perpetuate inequalities by shaping clinical decision-making [14,19]. Evaluation and mitigation of potential biases must occur throughout the model development life cycle to protect patients from algorithmic unfairness.

Reviewed studies frequently identified racial bias in clinical ML models. Notably, 1 algorithm in clinical use for prediction of future health care expenditures was found to discriminate against Black patients when compared to White patients, potentially contributing to disparities in health care delivery [36]. Other ML models that possibly demonstrate racial bias remain in preclinical states of development. Several studies have explicitly studied racial bias against Black patients compared to White patients. For example, 2 studies demonstrated that ML algorithms predicted similar risk scores in Black and White patients, though the Black patients were less healthy [36,37], and another demonstrated that an opioid misuse classifier had a higher FNR for Black patients [39]. Disparities in mortality prediction and X-ray diagnosis were identified in other races and ethnic groups [33,34,38], as well as disparities in burn identification and diabetic retinopathy identification in dark-skinned versus lighter-skinned patients [29,32]. Although conclusions cannot be drawn regarding the prevalence of racial bias among published clinical ML studies, the broad scope of clinical ML models susceptible to racial bias in this review exposes the potential of racial bias encoded in ML models to negatively impact patients across all aspects of health care.

### Assessment of Racial Bias

Clinical ML models must be carefully evaluated for potential biases imposed upon patients. Different fairness metrics may highlight different aspects of fairness relevant to a particular clinical setting; therefore, evaluation of all appropriate fairness metrics is needed when evaluating for potential bias. For example, calibration is particularly important to models performing risk prediction, while equal opportunity and disparate impact are relevant to screening and diagnostic settings. Inconsistent choice of fairness metrics among studies included in this review shows the need for a more standardized assessment process of racial bias in clinical ML. Some studies assessed fairness using metrics such as accuracy, area under the receiver operating characteristic curve (AUROC), and correlation of outcome with race, which may not sufficiently evaluate fairness [21]. Moreover, there are inherent trade-offs to the use of different fairness metrics [25], and static fairness criteria may even lead to delayed harms in the long term [43].

Obermeyer et al [36] present an example of using model calibration in conjunction with varied outcome labels to successfully de-bias an algorithm used to manage population health, and case studies have examined trade-offs of bias evaluation metrics in other settings, such as criminal justice [44], which may also serve as useful frameworks for clinical ML researchers. Use of “causal models,” which allow for closely tailored examination of discriminatory relationships in data, is another opportunity for investigation and mitigation of biased model behavior [45]. An increased focus from medical journals on bias evaluation checklists applicable to clinical ML models, such as the Prediction Model Risk of Bias Assessment Tool (PROBAST), is desirable to further emphasize vigilance regarding biased ML models [46]. Ultimately, more thorough analysis of fairness criteria in clinical ML will allow researchers to better contextualize and act on potential biases.

Clinical ML researchers should also be aware of potential barriers to ML fairness when adapting pretrained models and data representations. For instance, deep neural networks performing image processing tasks are frequently pretrained on large data sets and then fine-tuned to adapt to other tasks. Methods for removal of spurious variations from such models have been described, such as joint learning and unlearning algorithms, which account for contributions of undesirable variations during model development [47]. Language models trained in an unsupervised manner on vast amounts of text may learn biases present in training data [48]. Similarly, biases have been described in word embeddings [49], which are vectorized word representations used as inputs to ML models. Identification of bias in embeddings raises concerns about performance disparities in clinical applications of natural language processing if the bias is not screened for and appropriately addressed [50]. The lack of interpretability often inherent to ML models heightens the need for thorough evaluation of their potential biases.

### Creating Fair Models

Preprocessing and postprocessing methods of bias mitigation were successfully implemented among the publications reviewed for this study. Postprocessing methods appear to be easier to implement and may allow tailoring of imperfect models to new settings [51]. However, using preprocessing and in-processing to create unbiased data sets and algorithms at the outset of model development is desirable to facilitate the creation of fair, generalizable models. Continued evaluation of these techniques in clinical contexts is needed to inform best practices.

As data quality is generally the limiting factor to development of robust ML models, improvements to data generally translates directly into model performance improvements. Supplementation of data sets using generative models to synthesize patient data may be a viable approach to address data limitations. A study by Burlina et al [32] illustrated this fact by using a generative adversarial network to synthesize fundoscopy images while reducing class imbalance. However, though data limitations may contribute to disparities in model performance across racial groups, algorithmic unfairness may arise from other underlying biases in data as well [38]. Publications included in this review demonstrated improved fairness in ML models using multisource data sets, which may mitigate biases in the data collection process of single-source data sets [29,38]. Moreover, care must also be taken to ensure that multi-institutional data sets are appropriately prepared and used due to evidence that site-specific signatures contribute to bias in ML models [52]. Finally, protected attributes should not simply be ignored during model development, an approach called “fairness through unawareness,” as models may be able to infer protected group membership from other data features. Additionally, omission of protected attributes may cause bias if a legitimate relationship exists between the attribute and outcome of interest [19].

Several online resources aggregate examples and code implementations of published fairness evaluation and bias mitigation methods. Some examples of these resources include Aequitas, Artificial Intelligence Fairness 360 (IBM, Armonk, NY, United States), and Fairlearn (Microsoft Corporation, Redmond, WA, United States) [53,54]. Additionally, TensorFlow, a popular deep learning framework, includes a tool for evaluation of fairness indicators. Work by Briggs et al [31] highlights the feasibility and positive impact of standardized methodologies for addressing bias using a variety of performance indicators and mitigation techniques. Greater adoption of these and other strategies in fairness evaluation and bias mitigation will help set standard benchmarks for fairness in clinical ML.

### The Role of Transparency and Data Availability

ML is often characterized as a black box due to its limited interpretability, which is particularly problematic when attempting to address and prevent racial biases in clinical ML [55]. Although research in recent years has yielded significant progress in explainable ML methods [56], publication of model development code and data sets remains the most straightforward approach to transparency. Regrettably, medical ML research falls far short of these standards [57,58]. Code and data availability was inconsistent among the publications included in this review, and the majority of studies evaluated racial bias using publicly available data sets, including the Medical Information Mart for Intensive Care (MIMIC) [30,33,38], Kaggle EyePACS [32], and Dissecting Bias [31]. Considering the vast number of private, institutional data sets used to develop clinical ML models, there is a crucial need for future publications to maximize transparency, ensuring the ability to evaluate for fairness in clinical ML.

Increased publication of institutional data sets would facilitate the interdisciplinary collaboration needed to translate concepts of fairness in ML into the realm of medicine. Improved availability of data sets would also enable researchers to more easily validate existing models and perform fairness evaluations on different patient populations, translating benefits of ML across populations. Additionally, collaboration between institutions to maintain diverse, broadly representative data sets would facilitate the development of generalizable models free of the biases inherent to single-institutional data. However, ethical and patient confidentiality considerations may limit publication of clinical data. In contrast, publication of code and trained models, which are infrequently made available in the clinical ML literature [1,59], would similarly allow researchers to assess clinical ML on diverse populations without limitations imposed by patient privacy standards or institutional data-sharing regulations. Another possible paradigm to mitigate bias by training on diversely representative data sets while maintaining data privacy is federated learning, which involves piecewise training of an ML model on separate data sets and removes the need for data sharing during model development [60].

Moreover, increased emphasis on fairness in clinical ML through adoption of model development and reporting guidelines is needed [59,61]. Reporting guidelines for medical ML studies are inconsistently adopted, due in part to a lack of editorial policies among medical journals [1]. Moreover, reporting of demographic information needed to assess biases due to data sets is lacking [62,63]. The proposed Minimum Information for Medical AI Reporting guideline addresses these concerns by recommending that clinical ML studies report information necessary for understanding potential biases, including relevant demographic information of patient data used for model development [64]. In conjunction with upcoming reporting guidelines tailored to clinical ML [61], efforts to improve reporting quality will contribute to a standardized framework for fairness evaluation and bias mitigation in clinical ML.

### Limitations

As with any literature review, there are limitations to this study. Given the heterogeneity of terminology used to describe ML and racial bias, our search may have overlooked relevant publications. Additionally, we were limited by publication bias as we excluded publications other than full-length manuscripts, and researchers may be less likely to publish results confirming the absence of racial bias in a clinical ML model. Finally, the novelty of ML fairness in medicine and the resulting paucity of literature on this topic, as well as the breadth of relevant subjects encompassed, prevented us from obtaining the quantity and quality of data required to perform a systematic review or meta-analysis. In particular, the lack of standardized methods to evaluate and mitigate bias precludes any definitive conclusions regarding their suitability in clinical ML applications. However, the scoping review provides a methodological framework for critical evaluation of a previously uncharacterized area of research and draws attention to the lack of standardization regarding racial bias mitigation in clinical ML development. We emphasize the need for further work to build on this important aspect of the medical ML literature.

### Conclusion

Algorithmic fairness in clinical ML is a primary concern in its ethical adoption. As medical ML applications continue to approach widespread adoption across a multitude of clinical settings, potential racial biases in ML models must be proactively evaluated and mitigated in order to prevent patient harm and propagation of inequities in health care. The adoption of algorithmic fairness principles in medicine remains nascent, and further research is needed to standardize best practices for fairness evaluation and bias mitigation. We recommend that researchers and journal editors emphasize standardized reporting and data availability in ML studies to improve transparency and facilitate future research. Continued interrogation of biases in clinical ML models is needed to ensure fairness and maximize the benefits of ML in medicine.

## Appendix

Multimedia Appendix 1 Supplementary data file containing bias definitions, search strategy, and a table with study data set characteristics.

## Abbreviations

AI: artificial intelligence
AUROC: area under the receiver operating characteristic curve
FNR: false-negative rate
FPR: false-positive rate
LASSO: least absolute shrinkage and selection operator
ML: machine learning
PRISMA: Preferred Reporting Items for Systematic Reviews and Meta-analyses
TPR: true-positive rate
