# Supplementary material for: Evaluation and Mitigation of Racial Bias in Clinical Machine Learning Models: Scoping Review
Source: JMIR Med Inform. 2022 May 31;10(5):e36388. doi: 10.2196/36388 (PMC9198828; doi:10.2196/36388)
Supplement: Multimedia Appendix 1 [file medinform_v10i5e36388_app1.pdf]

Multimedia Appendix 1.

Bias Definitions

Term	Definition
Historical bias	Bias resulting from the propagation of existing real-world inequities into datasets.
Measurement bias	Also known as reporting bias; results from the selection, measurement, and utilization of particular features.
Representation bias	Bias resulting from the sampling method used to collect data on populations, leading to non-representative samples.
Temporal bias	Bias arising from changing behaviors and population characteristics over time.
Algorithmic bias	Bias added solely by an algorithm, independent of the data.
Population bias	Bias resulting from application of a model in a population that differs in characteristics from the original target or development population.
Aggregation bias	Bias occurring when conclusions drawn from a population are erroneously applied to individuals.

Definitions of common forms of bias relevant to clinical machine learning. For a more comprehensive overview, we recommend the following resource:

Mehrabi N, Morstatter F, Saxena N, Lerman K, Galstyan A. A survey on bias and fairness in machine learning. ACM Comput Surv 2021 Jul;54(6):1-35. [doi: 10.1145/3457607]

Database Search Queries

PubMed query

("artificial intelligence"[MeSH] OR "artificial intelligence*"[tiab] OR "machine learning*"[tiab] OR "deep learning*"[tiab] OR "supervised learning"[tiab] OR "unsupervised learning"[tiab] OR "neural network*"[tiab] OR "support vector machine*"[tiab] OR "markov decision process*"[tiab] OR "markov model*"[tiab] OR "natural language"[tiab] OR "decision tree*"[tiab] OR "k-nearest neighbor*"[tiab] OR "k nearest neighbor*"[tiab] OR "fuzzy c-mean*"[tiab] OR "fuzzy c mean*"[tiab] OR "deep-learning"[tiab] OR "deep feature"[tiab] OR "deep neural network*"[tiab] OR "deep belief network*"[tiab] OR "artificial neural network"[tiab] OR "convolutional neural network*"[tiab] OR "recurrent neural network*"[tiab] OR "feedforward neural network*"[tiab] OR "Boltzmann machine*"[tiab] OR "long short-term memory"[tiab] OR "long short term memory"[tiab] OR "gated recurrent unit"[tiab] OR "autoencoder"[tiab] OR "multilayer perceptron"[tiab] OR "convnet"[tiab] OR "convolutional learning"[tiab] OR "generative model"[tiab] OR "generative adversarial network"[tiab])
AND
("bias*"[tiab] OR "racis*"[tiab] OR "prejud*"[tiab] OR "fair*"[tiab] OR "equity"[tiab] OR "equitab*"[tiab] OR "unfair*"[tiab] OR "inequity"[tiab] OR "inequitab*"[tiab])
AND
("race"[tiab] OR "racial*"[tiab] OR "ethnic*"[tiab])

Embase query

('machine learning'/exp OR 'artificial intelligence*':ti,ab OR 'machine learning*':ti,ab OR 'deep learning*':ti,ab OR 'supervised learning':ti,ab OR 'unsupervised learning':ti,ab OR 'neural network*':ti,ab OR 'support vector machine*':ti,ab OR 'markov decision process*':ti,ab OR 'markov model*':ti,ab OR 'natural language':ti,ab OR 'decision tree*':ti,ab OR 'k-nearest neighbor*':ti,ab OR 'k nearest neighbor*':ti,ab OR 'fuzzy c-mean*':ti,ab OR 'fuzzy c mean*':ti,ab OR 'deep-learning':ti,ab OR 'deep feature':ti,ab OR 'deep neural network*':ti,ab OR 'deep belief network*':ti,ab OR 'artificial neural network':ti,ab OR 'convolutional neural network*':ti,ab OR 'recurrent

neural network*':ti,ab OR 'feedforward neural network*':ti,ab OR 'boltzmann machine*':ti,ab OR 'long short-term memory':ti,ab OR 'long short term memory':ti,ab OR 'gated recurrent unit':ti,ab OR 'autoencoder':ti,ab OR 'multilayer perceptron':ti,ab OR 'convnet':ti,ab OR 'convolutional learning':ti,ab OR 'generative model':ti,ab OR 'generative adversarial network':ti,ab)
AND
('bias*':ti,ab OR 'racis*':ti,ab OR 'prejud*':ti,ab OR 'fair*':ti,ab OR 'equity':ti,ab OR 'equitab*':ti,ab OR 'unfair*':ti,ab OR 'inequity':ti,ab OR 'inequitab*':ti,ab)
AND
('race':ti,ab OR 'racial*':ti,ab OR 'ethnic*':ti,ab)

Scopus query

(TITLE-ABS ("artificial intelligence*" OR "machine learning*" OR "deep learning*" OR "supervised learning" OR "unsupervised learning" OR "neural network*" OR "support vector machine*" OR "markov decision process*" OR "markov model*" OR "natural language" OR "decision tree*" OR "k-nearest neighbor*" OR "k nearest neighbor*" OR "fuzzy c-mean*" OR "fuzzy c mean*" OR "deep-learning" OR "deep feature" OR "deep neural network*" OR "deep belief network*" OR "artificial neural network" OR "convolutional neural network*" OR "recurrent neural network*" OR "feedforward neural network*" OR "Boltzmann machine*" OR "long short-term memory" OR "long short term memory" OR "gated recurrent unit" OR "autoencoder" OR "multilayer perceptron" OR "convnet" OR "convolutional learning" OR "generative model" OR "generative adversarial network"))
AND
(TITLE-ABS ("bias*" OR "racis*" OR "prejud*" OR "fair*" OR "equity" OR "equitab*" OR "unfair*" OR "inequity" OR "inequitab*"))
AND
(TITLE-ABS ("race" OR "racial*" OR "ethnic*"))

Google Scholar query

"racial bias artificial intelligence health"

Study Dataset Characteristics and Code Availability

Author	Year	Country	Dataset size	Data source	Model type	Code availability
Abubakar et al.	2020	United Kingdom, Nigeria	1360 (Caucasian), 540 (African)	Institutional data from two hospitals	ResNet50 (CNN)	No
Allen et al.	2020	United States	28,460	MIMIC-III	XGBoost	Yes (data reweighting)
Briggs & Hollmén	2020	Sweden	48,784	Dissecting Bias	Unspecified; classification algorithms were selected per AI Fairness 360 examples	Yes
Burlina et al.	2021	United States	21,720	Kaggle EyePACS	ResNet50 (CNN)	No
Chen et al.	2019	United States	25,879 (ICU notes); 4,214 (psychiatric notes)	MIMIC-III; single-institutional data	Logistic regression	Yes (data selection, bias analysis)
Gianattasio et al.	2020	United States	835	Aging Demographics and Memory Study	Logistic regression, LASSO	Yes (develop-ment)
Noseworthy et al.	2020	United States	97,829	Single-institutional data	CNN	Request

Obermeyer et al.	2019	United States	49,618	Single-institutional data	LASSO	Yes (develop-ment, bias analysis)
Park et al.	2021	United States	573,634	IBM Marketscan Medicaid Database	Logistic regression, random forest, XGBoost	No
Seyyed-Kalantari et al.	2021	Canada	129,819	MIMIC-CXR	DenseNet (CNN)	No
Thompson et al.	2021	United States	53,974	Institutional data from two hospitals	CNN	Yes (develop-ment)
Wissel et al.	2019	United States	3,776	Single-institutional data	SVM	No

Supplementary Table 1: Study dataset characteristics and code availability. CNN: convolutional neural network; XGBoost: extreme gradient boosting; AI: artificial intelligence; ICU: intensive care unit; LASSO: least absolute shrinkage and selection operator; AUC: area under the receiver operating characteristic curve; SVM: support vector machine
